# Development of EST-SSRs based on the transcriptome of *Castanopsis carlesii* and cross-species transferability in other *Castanopsis* species

**DOI:** 10.1371/journal.pone.0288999

**Published:** 2023-07-20

**Authors:** Xiaoru Zhong, Mengyang Xu, Ting Li, Rongxi Sun

**Affiliations:** Jiangxi Provincial Key Laboratory of Silviculture, College of Forestry, Jiangxi Agricultural University, Nanchang, China; Nuclear Science and Technology Research Institute, ISLAMIC REPUBLIC OF IRAN

## Abstract

*Castanopsis carlesii* (Hemsl.) Hay. is a widely distributed and dominant tree species native to subtropical China with significant ecological and economic value. Due to serious human-related disturbance, its wild resources have been increasingly reduced, and whether may result in the loss of genetic diversity. However, no population genetics studies of natural *C*. *carlesii* have been reported to date. Microsatellite markers have been a useful tool in population genetics. Therefore, we developed EST-SSR markers based on the transcriptome sequencing of *C*. *carlesii* leaves. A total of 149,380,224 clean reads were obtained, and 63,012 nonredundant unigenes with a mean length of 1,034 bp were assembled and annotated based on sequence similarity searches in the Nr, Nt, KO, SwissProt, PFAM, KOG, and GO databases. The results showed that only 5,559 (8.82%) unigenes were annotated in all seven databases, but 46,338 (73.53%) could be annotated in at least one database. A total of 31,459 potential EST-SSRs were identified in 18,690 unigenes, with an average frequency of one SSR approximately 2 kb. Among the 100 EST-SSR primer pairs designed, 49 primer pairs successfully produced the expected product by amplification, with a success rate of 49%, but only 20 primer pairs showed abundant polymorphisms. Polymorphisms were verified using 25 samples from *C*. *carlesii* in Qimen, Anhui. A total of 119 alleles were detected, with a mean number of alleles (*Na*) of 5.95 per locus and a mean polymorphism information content (*PIC*) of 0.6125. All the 20 newly developed EST-SSR markers were verified in other *Castanopsis* species (*C*. *sclerophylla*, *C*. *lamontii*, *C*. *fargesii*, *C*. *eyrei* and *C*. *jucunda*). Sixteen primer pairs showed successful amplification in all five *Castanopsis* species (80%), and the transferability ratios ranged from 90% to 100%. These developed EST-SSR markers can be applied to population genetic and germplasm evaluations of *C*. *carlesii* and related species.

## Introduction

*Castanopsis carlesii* (Hemsl.) Hay. is a widely distributed and dominant tree species belonging to the family Fagaceae in subtropical evergreen broadleaved forests of China, with significant economic and ecological value [1]. It is valuable for high quality wood and edible nuts with high starch and soluble sugar contents. The wood is durable, strong, and resistant to decay, making it is good timber species for use in construction, furniture, and other wood products. The nuts are a traditional natural food with rich in trace elements. Furthermore, the species has considerable ecological functions, such as contributing to carbon sequestration, soil and water conservation, and disaster prevention and mitigation. However, subtropical forests have been severely fragmented and deforested due to human disturbance and land use change over the last century, Large areas of evergreen broadleaved forests have been transformed to farmland or plantations [2]. Hence, wild resources of *C*. *carlesii* in subtropical regions have been increasingly reduced, and whether may result in the loss of genetic diversity. The loss of genetic diversity might lead to decline in species adaptability and evolution. Therefore, evaluating the genetic diversity of *C*. *carlesii* resources could aid in protecting the genetic diversity of this species and formulating scientific management strategies.

Next-generation sequencing (NGS) technology has become a cutting-edge method for obtaining a large amount of genomic data. Transcriptome sequencing is both feasible and affordable [3–5]. In particular, transcriptome sequencing facilitates the discovery and exploration of molecular markers for nonmodel pants [6,7]. In addition, it can be applied for analyses of functional genes and differential gene expression patterns and transcriptome profiling [8,9]. Furthermore, NGS technology has been widely used in conservation genetics and evolution research by producing large scale informative markers at the genome and transcriptome levels.

Compared with other types of molecular markers such as intersimple sequence repeat markers (ISSRs), amplified fragment length polymorphisms (AFLPs) and random amplification of polymorphic DNAs (RAPDs), simple sequence repeats (SSRs) are more efficient due to their codominance, higher reproducibility and cross-species transferability [10], which indicates that SSR markers play an important role in genetic diversity and marker assisted selection research [11,12]. SSRs can be divided into expressed sequence tag SSRs (EST-SSRs), identified in transcribed RNA sequences, and genomic SSRs (g-SSRs), identified in genomic sequences [13]. EST-SSRs exhibit excellent interspecific transferability, a lower frequency of null alleles, and greater evolutionarily conservation relative to g-SSRs [14].

In this study, the transcriptome sequencing of *C*. *carlesi* was conducted on the Illumina sequencing platform. Unigenes obtained from transcriptome data were assembled and annotated. EST-SSR markers were developed based on the transcriptome data, and polymorphisms were evaluated in a representative population. Additionally, their cross-species transferability was examined in related species of the *Castanopsis* genus.

## Materials and methods

### Plant materials, RNA and DNA extraction

Fresh young leaves of 3 *C*. *carlesii* accessions to be used for RNA isolation were collected from the Jiulianshan National Nature Reserve in Ganzhou of Jiangxi Province, China (24°33′N, 114°25′E), which was approved by the Administration of Jiangxi Jiulianshan National Nature Reserve. The collected young leaves were frozen in liquid nitrogen and then stored at −80°C until RNA extraction. RNA degradation and contamination were detected on 1% agarose gels. The purity and integrity of RNA were checked on a Nano Photometer spectrophotometer (IMPLEN, CA, USA) and an Agilent Bioanalyzer 2100 system (Agilent Technologies, CA, USA), respectively. Approximately 1.5 μg of RNA from three individuals, pooled in equal amounts, was used for cDNA library construction.

Fresh young leaves to be used for DNA isolation were collected in silica gel from different locations in subtropical China (S1 Table). Total genomic DNA was isolated using a Plant Genomic DNA Kit (TIANGEN, Beijing, China). The quality and concentration of the DNA were determined by electrophoresis on a 0.8% agarose gel and with a spectrophotometer (Eppendorf, Hamburg, Germany), respectively. The DNA samples were diluted to a working concentration of 50 ng/μL for a subsequent experiment.

### Transcriptome assembly and sequence data analysis

Raw data were first processed through Perl scripts. Clean data were obtained by removing reads containing adapters and ploy-N sequences (>10%) and reads with low quality from the raw data. At the same time, the Q20, Q30, GC content and sequence duplication levels of the clean data were computed. Transcriptome assembly was accomplished using Trinity [15] with min_kmer_cov set to 2 by default and all other parameters set to the defaults. The assembled transcripts were compared by BLASTX in public databases (E-value ≤ 10^−5^), such as NR (NCBI nonredundant protein sequences), SwissProt (a manually annotated and reviewed protein sequence), KEGG (Kyoto Encyclopedia of Genes, and Genomes), and PFAM (Protein family) [16]. Blast2GO was used to provide insight into GO (Gene Ontology) annotations including biological process, cellular component ontologies and molecular function categories [17]. In additon, the KOG (euKaryotic Orthologous Groups of proteins) database was used to categorize and predict their possible functions.

### SSR marker development

Microsatellite loci were detected using MISA (https://webblast.ipk-gatersleben.de/misa/) [18] according to the following criteria: mono-nucleotide repeat motifs with at least 10 repeats, di-nucleotide repeat motifs with at least six repeats, tri-nucleotide, tetra-nucleotide, penta-nucleotide and hexa-nucleotide repeat motifs with at least five motifs. Primer pairs flanking the SSRs were designed with Primer3 [19] according to the following core criteria: the optimal primer length was 20 bp, ranging from 18 to 22 bp; the GC content ranged from 40% to 60% and the amplified products were 100–300 bp in size. Mono-nucleotide repeat motifs were excluded in the synthesized SSR primers for their higher likelihood of errors during PCR amplification. The primers were synthesized by RuiBiotech (Beijing, China).

Gene coding structure was searched using of the Trinity transdecoder module to gain insight into the distribution of SSRs in the *C*. *carlesii* leaf transcriptome, which indicated SSR loci located in 5’ untranslated regions (5’ UTRs), 3’ untranslated regions (3’UTRs) or coding DNA sequence (CDS) regions [20]. In particular, SSRs located in CDS regions are highly desirable as they can potentially have functional significance and contribute to phenotypic variation. While, SSRs located in UTRs are also important as they can affect gene expression and regulation. One hundred pairs of SSR primers were tested in 8 individuals collected from 8 different populations (S1 Table) to verify their polymorphism and suitability. The M13-tail polymerase chain reaction (PCR) method of Schuelke was used to measure the size of the PCR products [21]. The method involves three primers: a forward SSR specific primer with an M13 (5’- TGTAAAACGACGGCCAGT-3’) tail at the 5’ end labeled with a phosphoramidite fluorescent dye-labeled (ROX, TAMRA, FAM or HEX), a reverse SSR specific primer, and the M13 (5’-TGTAAAACGACGGCCAGT-3’) universal primer. PCR amplification was performed in two steps. The first PCR amplification was performed in a 10 μL volume containing approximately 50 ng DNA, 1× Taq mastermix, 10 μM forward primer with the M13 tail and 10 μM reverse primer, and sterile double-distilled water was added to a final volume of 10 μL. The first PCR amplification was performed under the following conditions: 95°C for 5 min, followed by 20 cycles of 95°C for 30 s, annealing at optimal temperature for 30 s and 72°C for 30 s, with a final extension at 72°C for 10 min. The second amplification was performed in a 20 μL volume containing 2 μL PCR products from the first PCR amplification, 1× Taq mastermix, 10 μM reverse primer and 10 μM fluorescently labeled M13 primer, with sterile double-distilled water added to 20 μL. The second PCR amplification was performed at 95°C for 5 min, followed by 35 cycles of 95°C for 30 s, 52°C for 30 s and 72°C for 30 s, with a final extension at 72°C for 10 min. PCR products were sequenced on an ABI 3730XL capillary electrophoresis analyzer (Applied Biosystems, Foster, CA, USA). GeneMaker 2.2.0 software was used for fragment genotyping.

### Genetic diversity analysis and cross-species amplification

The number of alleles (*Na*), number of effective alleles (*Ne*), information index (*I*), observed heterozygosity (*Ho*), and expected heterozygosity (*He*) were calculated by POPGENE32 software [22]. The polymorphic information content (*PIC*) was computed using CERVUS version 3.0 [23]. Five species of the genus *Castanopsis* (*C*. *sclerophylla*, *C*. *lamontii*, *C*. *fargesii*, *C*. *eyrei* and *C*. *jucunda*) were used to evaluate the transferability of these newly developed EST-SSR markers.

## Results

### Transcriptome sequencing and de novo assembly

In total, 153,146,384 raw sequence paired-end reads were obtained using the HiSeq2500 platform. All raw reads have been deposited in the National Center for Biotechnology Information Sequence Read Archive (NCBI SRA with accession number PRJNA907071). After trimming contaminated sequences and removing low-quality reads, 149,380,224 clean reads with an average GC content of 44.37% were obtained and used for de novo assembly. A total of 22.41 G were obtained, and the Q20 and Q30 values were 97.68% and 93.43%, respectively. A total of 63,012 unigenes were obtained, ranging in size from 301 to 15,700 bp, with a total length of 65,128,186 bp. Among these unigenes, the mean length was 1034 bp, and the N50 and N90 values were 1,427 bp and 476 bp, respectively. A total of 164,384 transcripts with 167,759,847 nucleotides were obtained with a mean length of 1,021 bp, and the N50 and N90 values were 1,518 bp and 429 bp, respectively (Table 1). Among the 63,012 unigenes, 19,087 (30.29%) were between 301 and 500 bp, 23,059 (36.60%) were between 501 and 1,000 bp, 13,315 (21.13%) were between 1,001 and 2,000 bp, and 7,551 (11.98%) had a length > 2,000 bp. Among these 64,384 transcripts, 61,978 (37.70%) had lengths ranging from 301 to 500 bp, 46,780 (28.46%) from 501 to 1,000 bp, 35,009 (21.30%) from 1,001 to 2,000 bp, and 20,617 (12.54%) had lengths longer than 2,000 bp (S1 Fig).

**Table 1:** De novo assembly of *C*. *carlesii* sequences.

Category	Items	Number
**Raw reads**	Total raw reads	153,146,384
**Clean reads**	Total clean reads	149,380,224
	Q20 percentage	97.68%
	Q30 percentage	93.43%
	GC content	44.37%
**Unigenes**	Total sequence number	63,012
	Total nucleotides	65,128,186
	Max length	15,700
	Min length	301
	Mean length	1,034
	N50 (bp)	1,427
	N90 (bp)	476
**Transcripts**	Total sequence number	164,384
	Total nucleotides	167,759,847
	Max length	15,700
	Min length	301
	Mean length	1,021
	N50 (bp)	1,518
	N90 (bp)	429

### Functional annotation and classification

All (63,012) unigenes were annotated by sequence similarity searching in seven public databases as described in the method. A total of 35,366 (56.12%) unigenes were aligned to the NR database, with 27,238 (43.22%) unigenes showing significant similarity to nonredundant nucleotide sequences in the NT sequence database, while, 14,134 (22.43%) were annotated in the KO database, 31,579 (50.11%) in the SwissProt database, 30,570 (48.51%) in the PFAM and GO databases and 12,701 (20.15%) in the KOG database. Only 5,559 (8.82%) unigenes were annotated in all seven databases, but 46,338 (73.53%) could be annotated in at least one database (S2 Fig, S2 Table).

According to the species statistics based on the NR database, 38,795 (89.7%) genes were annotated to *Quercus suber*, 970 (2.2%) genes were annotated to *Juglans regia*, 276 (0.6%) genes were annotated to *Hordeum vulgare*, and 180 (0.4%) and 120 (0.3%) genes were annotated to *Vitis vinifera* and *Coccomyxa subellipsoidea*, respectively. This suggests that *C*. *carlesii* was closely related to *Q*. *suber* (S3 Fig).

**Fig 1:**
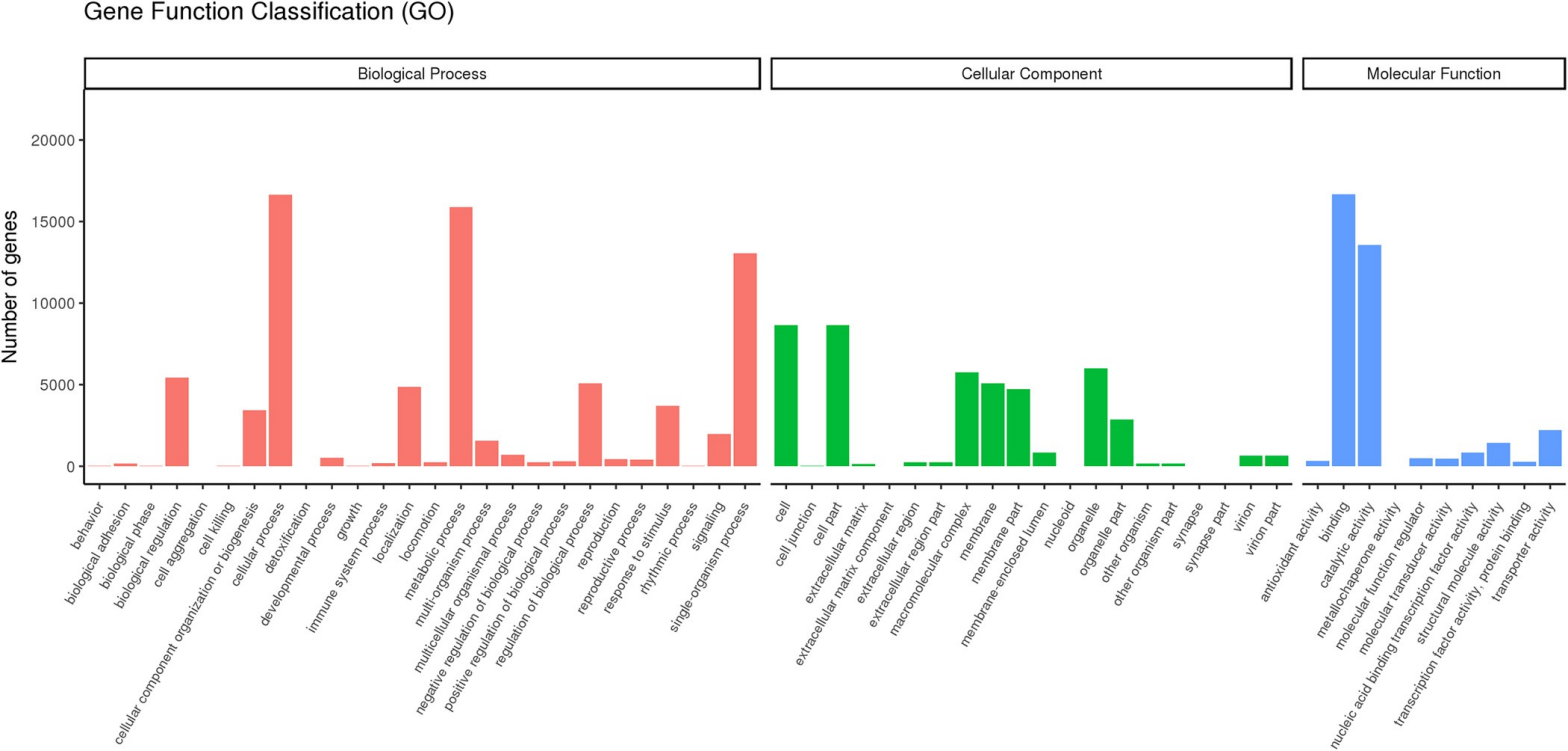
Summary of Gene Ontology (GO) analysis of *C*. *carlesii*. The x-axis indicates the number of unigenes, and the y-axis indicates a specific functional cluster.

A total of 30,570 unigenes were assigned to the biological process, cellular component and molecular function Gene Ontology (GO) categories, including 56 subcategories (Fig 1). In the biological process category, the cellular process (16,337, 54.42%), metabolic process (15,892, 51.99%), and single-organism process (13,060, 42.72%) terms were the most enriched of the 26 subgroups. Within the cellular component category, the cell (28.28%) and cell part (28.28%) terms, both inculding 8,646 unigenes, were the most enriched of all the 20 subgroups. Among the last 10 subgroups in the molecular function category, the binding (16,663, 54.51%) and catalytic activity (13,552, 44.33%) were the most enriched. However, only 2 unigenes each were assigned to cell aggregation, in the biological process category, and nucleoid, in the cellular component category (Fig 1, S2 Table).

**Fig 2:**
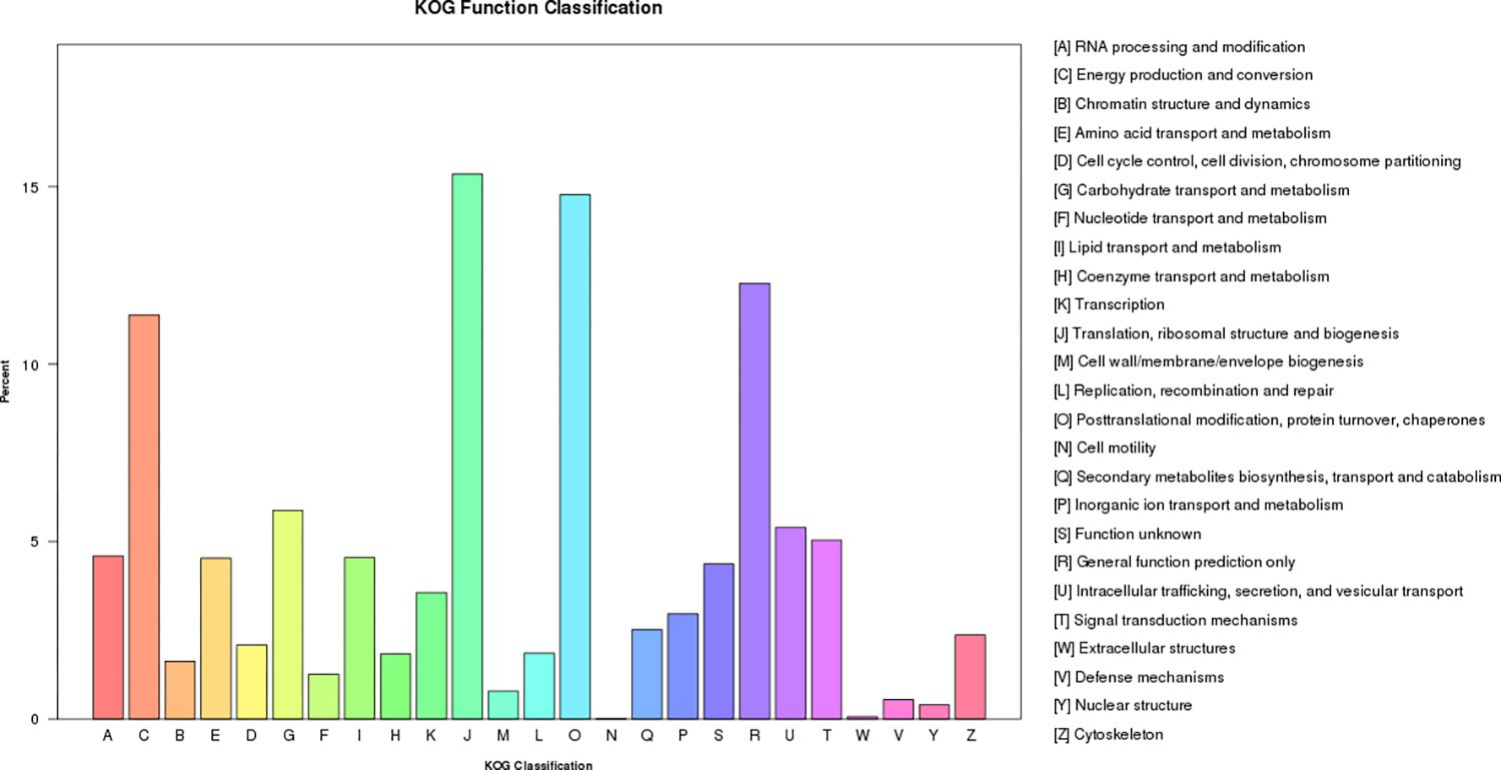
euKaryotic Orthologous Groups (KOG) classification of *C*. *carlesii*. The x-axis indicates the KOG classification. The right y-axis indicates the percentage of a specific category of genes and the number of genes in a category. The left y-axis indicates the percentage of a specific category of genes in the KOG category.

**Fig 3:**
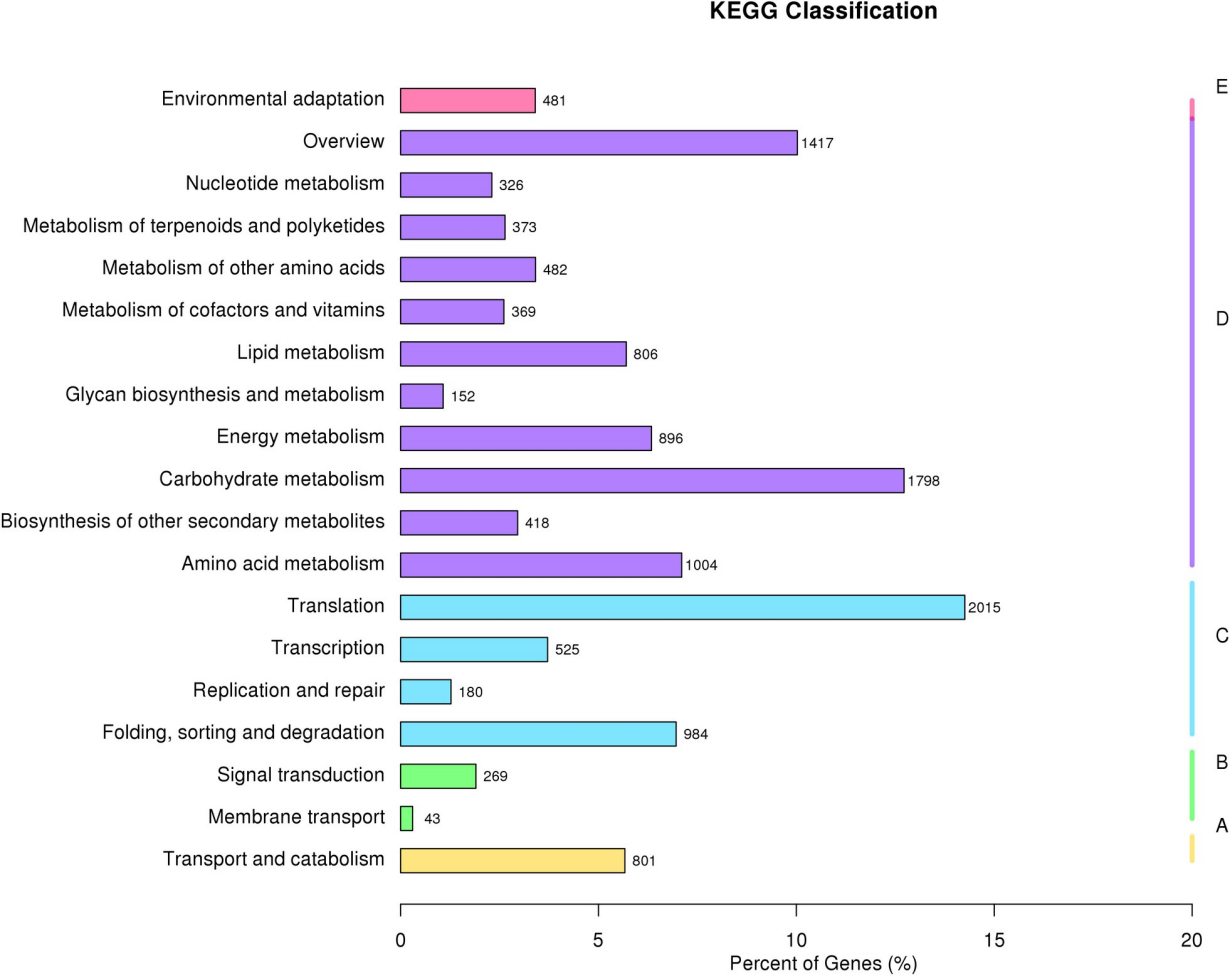
Kyoto Encyclopedia of Genes and Genomes (KEGG) metabolic pathway analysis of *C*. *carlesii*. The x-axis indicates the percentage of genes, and the y-axis indicates the KEGG metabolic pathway.

A total of 12,701 unique sequences were assigned to 25 functional KOG categories. Most unigenes fell within the “translation, ribosomal structure and biogenesis” category (1,950, 15.35%), followed by the “posttranslational modification, protein turnover, chaperones” (1,876, 14.77%) and “general function prediction only” (1,559, 12.27%) categories. In contrast, the “cell motility” category contained only three unigenes (0.02%) (Fig 2, S3 Table). KEGG pathway analysis showed that metabolism was the most enriched category (8,041, 60.28%), followed by genetic information processing (3,704, 27.77%), cellular processes (801, 6.00%), organismal systems (481, 3.61%), and environmental information processing (312, 2.34%). Among the 19 subgroups, translation (2,015) and carbohydrate metabolism (1,798) were the most represented pathways (Fig 3, S4 Table).

### EST-SSR identification

A total of 63,012 unigenes with a total length of 65,128,186 bp identified in *C*. *carlesii* were searched for the presence of EST-SSRs, and 31,459 potential EST-SSRs and 20,055 gene-based SSR marker were identified in 18,690 unigenes, with an average of approximately one SSR site per 2 kb. Among these SSRs, 40.26% (7,525) contained more than one SSRs (S5 Table). Mono-nucleotide were the most common type of repeat motif (16,594, 53.90%), followed by di-nucleotide (9,446, 30.03%) and tri-nucleotide (4,472, 14.22%). The three predominant motif types represented 98.13% of all SSRs, whereas only 297 (0.94%), 95 (0.30%) and 195 (0.62%) SSRs were tetra-nucleotide, penta-nucleotide and hexa-nucleotide repeat motifs, respectively. The number of SSR motif repeats varied from 5 to 93, among which the most common number was ten tandem repeats (5,018, 15.95%) (Table 2).

**Table 2:** Distribution and frequency of EST- SSRs in the *C*. *carlesii* transcriptome.

Repeats	5	6	7	8	9	10	11	12	>12	Total	Percentage (%)
**Mono-nucleotide (16,594)**											53.90
**A/T**	-	-	-	-	-	3,969	2,444	1,730	8,447	16,590	52.74
**C/G**	-	-	-	-	-	52	42	46	224	364	1.16
**Di-nucleotide (9,446)**											30.03
**AC/GT**	-	406	254	194	114	94	77	42	172	1,353	4.30
**AG/CT**	-	1,237	773	683	647	494	398	318	1673	6,223	19.78
**AT/AT**	-	264	174	155	182	239	242	217	364	1837	5.84
**CG/CG**	-	17	10	5		1			0	33	0.10
**Tri-nucleotide (4,472)**											14.22
**AAC/GTT**	265	133	102	52	29	15	2	1	2	601	1.91
**AAG/CTT**	532	290	183	116	73	85	12	16	31	1,338	4.25
**AAT/ATT**	241	122	56	34	41	39	8	7	25	573	1.82
**ACC/GGT**	256	117	80	41	17	8	2		0	521	1.66
**ATC/ATG**	210	107	40	23	15	9	2		0	406	1.29
**Others**	539	278	119	54	26	12	2	2	1	1,033	3.28
**Tetra-nucleotide (297)**											0.94
**AAAC/GTTT**	22	4	2						0	28	0.09
**AAAG/CTTT**	49	13	8						0	70	0.22
**AAAT/ATTT**	63	18	1							82	0.26
**ACAT/ATGT**	16	5	5			1				27	0.09
**AGAT/ATCT**	7	3	2							12	0.04
**Others**	57	16	5							78	0.25
**Penta-nucleotide (95)**											0.30
**AAAAC/GTTTT**	8	1								9	0.03
**AAAAG/CTTTT**	16	1								17	0.05
**AAAAT/ATTTT**	6	1								7	0.02
**Others**	52	10								62	0.20
**Hexa-nucleotide (195)**											0.62
**AACCAC/GGTTGT**	4	1	1	1						7	0.02
**AACACC/GGTGTT**	6	1								7	0.02
**AAGATG/ATCTTC**	6	1								7	0.02
**Others**	161	8	3	1	1					174	0.55
**Total**	2,516	3,054	1,818	1,359	1,145	5,018	3,231	2,379	10,939	31,459	
**Percentage (%)**	8.00	9.71	5.78	4.32	3.64	15.95	10.27	7.56	34.77		

Mono-nucleotide A/T repeat motifs were the highest number among all the SSRs, amounting to 16,590 (52.74%). Among the dinucleotide repeat motifs, AG/CT repeats were the most frequent (6,223, 19.78%), followed by AT/AT repeats (1,837, 5.84%) and AC/GT repeats (1,353, 4.30%). However, only 33 (0.10%) of these motifs were CG/CG repeats. Additionally, AAG/CTT repeats were the most common type (1,338, 4.25%) of tri-nucleotide repeat motifs. AAAT/ATTT and AAAAG/CTTTT were among the dominant tetra-nucleotide and penta-nucleotide repeat motifs, respectively (Table 2).

### EST‑SSR polymorphism validation

Among the 100 EST-SSR primer pairs designed, 49 primer pairs successfully produced the expected and clear amplicons, but only 20 primer pairs showed abundant polymorphisms (S6 Table). The characteristics of these 20 EST-SSR polymorphism markers were summarized in Table 3. Analysis of the primer locations revealed that 5 loci (20%) were located in coding DNA sequence (CDS) regions, 2 loci were located in the 3′ untranslated regions (3′-UTRs), and the remaining 13 loci were located in 5′ untranslated regions (5′-UTRs). The majority (80%) were located in the 3′-UTR or 5′-UTR, because these untranslated regions tend to have higher genetic variation than the coding DNA sequence (CDS) regions [24].

**Table 3:** Summary of 20 EST-SSR markers used for polymorphism analysis. Number of alleles (*Na*), number of effective alleles (*Ne*), Shannon’s information index (*I*), observed heterozygosity (*Ho*), expected heterozygosity (*He*), and polymorphism information content (*PIC*).

	ID	Sequence (5’-3’)	Motif	Position	Tm(°C)	Obs. Size range	*Na*	*Ne*	*I*	*Ho*	*He*	*PIC*
**1**	CC07	F:GGCACCATGGAAGCTCTCAA R:GGGGCTAGAGATCATTGGCC	(TG)12	CDS	60	218–240	8	6.4908	1.9521	0.9565	0.8647	0.827
**2**	CC17	F:AGGAGATGAAGGCCAGCAAC R:GCTCCCAATCCCATAGAGGC	(AAG)9	CDS	60	260–266	3	2.0799	0.8596	0.6800	0.5298	0.444
**3**	CC24	F:TGCCCGCAAGATCGTTTACT R:TTTTTGGTTGTGCCGCACTC	(ATG)9	CDS	60	205–214	4	2.4272	1.0142	0.5600	0.6000	0.502
**4**	CC32	F:CTCCGAATCCGAACCCAACA R:ACTCTGCTTTGGCTCTTGCT	(CAA)9	CDS	60	222–243	5	2.6005	1.1589	0.3750	0.6285	0.559
**5**	CC33	F:ATCGAATCGATCCGTGGTCG R:CCTTGCAACCCCCAAACATG	(AGA)11	CDS	60	175–193	6	3.7879	1.4564	0.7600	0.7510	0.691
**6**	CC45	F:ACAGTGTGTTTGACAAAACCTCA R:AGCTGTTCAGAACCGGATGG	(CT)10	3′-UTR	60	200–220	6	2.9138	1.3141	0.6000	0.6702	0.608
**7**	CC57	F:GGAGACTCTCGTCGTTGTGG R:TAACTCCGGCAATTGACCCC	(ACC)7	3′-UTR	60	174–180	2	1.3172	0.4050	0.2000	0.2457	0.212
**8**	CC63	F:CGAAGTCCCACAGAGCTGAG R:GATGAGCCTCGAGTAGCAGC	(GTTG)6	5′-UTR	60	208–224	4	3.6357	1.3367	0.7391	0.7411	0.675
**9**	CC65	F:AAATTTGTCCAGCCGGTCCA R:GCGATCCCAGCAGAATCGTA	(AGA)9	5′-UTR	60	224–266	12	6.0096	2.0677	0.8400	0.8506	0.816
**10**	CC69	F:GAGGATTACGGGATGGGCTG R:ACCTTGGCTATCGACGCATT	(AG)15	5′-UTR	60	121–147	8	3.0414	1.5281	0.6400	0.6849	0.648
**11**	CC72	F:GAGAGAAAGTGGTGACCGCA R:CCCCTTCTCCGTGGTGTTTT	(TTC)10	5′-UTR	60	168–180	5	3.5311	1.4069	0.6800	0.7314	0.637
**12**	CC80	F:GCTTTTGTGGAGCCAACAGG R:CACAGTGTTTTTGGCCCGAG	(ACT)8	5′-UTR	60	203–237	8	5.1653	1.785	0.9200	0.8229	0.780
**13**	CC84	F:TCATCGTTGTCGGTGGGAAG R:TCGATCGGTGTTGGAAGCAA	(AAG)7	5′-UTR	60	230–257	7	2.3408	1.2075	0.4800	0.5845	0.539
**14**	CC85	F:TTCCCTGGGATCATTTGGGC R:ACGCTTGATTTCAATGCTTCTCA	(ACT)13	5′-UTR	60	276–294	6	2.5773	1.2069	0.6400	0.6245	0.563
**15**	CC86	F:TGCCGGTTCGTATAGCTGTC R:AACGGCGTTTGAGTTGTTCG	(ATAG)6	5′-UTR	60	227–239	4	2.4894	1.0874	0.6522	0.6116	0.543
**16**	CC88	F:GGATTTGAGGTGGGTCTCGG R:ATTGAGTCCCAAAACCCGCA	(CCTGTG)6	5′-UTR	60	168–188	5	3.9862	1.4562	0.3750	0.7651	0.705
**17**	CC91	F:GCGTTTGCAGAAAGGGGTTT R:TCCACTTCCCATGCCAACTC	(GAG)7	5′-UTR	60	174–183	4	1.5188	0.6800	0.4000	0.3486	0.318
**18**	CC94	F:CTTGGAAACTGCGCCAACAA R:GTGGGGTGAGTTTGGGAGAG	(AGA)10	5′-UTR	60	148–172	8	4.562	1.7612	0.6800	0.7967	0.755
**19**	CC96	F:CGAGCAGCAACTTCTTCTTCC R:CGGAGCTGTGGTTGTGTTTG	(CTT)7	5′-UTR	60	136–154	5	2.8881	1.2402	0.1500	0.6705	0.601
**20**	CC98	F:GGTCATTGTAGAGGAGGGGG R:AACGGTGCCACAAGGGAATT	(TTC)13	5′-UTR	59	266–305	9	6.4908	1.9846	0.6087	0.8647	0.827
**Mean**							5.95	3.4927	1.3454	0.5968	0.6694	0.6125

The polymorphisms of 20 EST-SSR loci were verified using 25 samples from *C*. *carlesii* in Qimen, Anhui. A total of 119 alleles were detected in all samples based on the 20 EST-SSR loci (Table 3). The number of alleles (*Na*) per locus varied from 2 (CC57) to 12 (CC65), with a mean of 5.95, and the effective number of alleles (*Ne*) varied from 1.3172 (CC57) to 6.4908 (CC07 and CC98), with a mean value of 3.4927. Shannon’s information index (*I*) showed an average value of 1.3454 and varied between 0.4050 (CC57) and 2.0677 (CC65). The ranges of observed (*Ho*) and expected (*He*) heterozygosity were from 0.1500 (CC96) to 0.9565 (CC07) and 0.2457 (CC57) to 0.8647 (CC98), with average values of 0.5968 and 0.6694, respectively. The polymorphism information content (*PIC*) ranged from 0.212 (CC57) to 0.827 (CC07 and CC98), with an average of 0.6125. The *PIC* values indicated that 17 loci, accounting for 85% of the markers, were highly polymorphic (*PIC*>0.5), and only 2 loci (CC17 and CC91) and 1 locus (CC57) presented moderate polymorphism (0.25<*PIC*<0.5) and low polymorphism (*PIC*<0.25), respectively. These results suggested that the majority of the EST-SSR markers developed were highly informative and have the potential to be used for population studies.

### Cross-species transferability of EST-SSR markers

To estimate cross-species transferability, all the 20 newly developed EST-SSR markers were verified in other *Castanopsis* species (*C*. *sclerophylla*, *C*. *lamontii*, *C*. *fargesii*, *C*. *eyrei* and *C*. *jucunda*). Sixteen primer pairs (80%) showed successful amplification in all five *Castanopsis* species, indicating that they have good cross-species transferability. Partial capillary electrophoresis profiles of primer pairs CC57, CC45 and CC86 were shown in S4 Fig. All the 20 primer pairs were applicable to *C*. *eyrei* and *C*. *jucunda*, with the highest transferability rate of 100%. Additionally, we confirmed that 19 (95%) and 18 (90%) primer pairs were applicable to *C*. *sclerophylla*, *C*. *fargesii and C*. *lamontii*, respectively (Table 4). The transferability ratios ranged from 90% to 100% which indicating that these markers have broad applications for studying genetic diversity in different *Castanopsis* species.

**Table 4:** Transferability across five *Castanopsis* species of the EST-SSR markers validated in this study.

Loci	*C*. *sclerophylla*	*C*. *lamontii*	*C*. *fargesii*	*C*. *eyrei*	*C*. *jucunda*
**CC57**	+	+	+	+	+
**CC45**	+	+	+	+	+
**CC86**	+	−	+	+	+
**CC69**	+	+	+	+	+
**CC33**	+	+	+	+	+
**CC32**	+	−	+	+	+
**CC72**	+	+	+	+	+
**CC07**	+	+	+	+	+
**CC17**	+	+	+	+	+
**CC88**	+	+	+	+	+
**CC80**	+	+	+	+	+
**CC98**	−	+	+	+	+
**CC91**	+	+	+	+	+
**CC63**	+	+	+	+	+
**CC85**	+	+	+	+	+
**CC94**	+	+	+	+	+
**CC65**	+	+	+	+	+
**CC96**	+	+	−	+	+
**CC24**	+	+	+	+	+
**CC84**	+	+	+	+	+
**Transferability rate (%)**	95	90	95	100	100

A plus sign indicates that the primer was successfully amplified in this species, whereas a minus sign indicates that the primer failed to generate clear PCR products. Gray shading indicates that the primer showed successful amplification in all five *Castanopsis* species.

## Discussion

### Characterization of the *C*. *carlesii* transcriptome

In nonmodel plants without reference genomes, transcriptome sequencing is regared as the most effective way of identifying functional genes and developing novel molecular markers to identify functional genes and develop molecular markers [25,26]. In the present evaluation, the transcriptome of *C*. *carlesii* was initially documented by utilizing Illumina sequencing technology. A total of 149,380,224 clean reads were generated, with a 97.68% Q20 level and 93.43% Q30 level, respectively. The N50 values and the mean lengths of the unigenes were 1,427 bp and 1,034bp, respectively, which were greater than the values obtained in previous transcriptomic (leaf tissue) analyses of Fagaceae family members, such as *Quercus austrocochinchinensis* (N50 = 1,335 bp, 782 bp) and *Quercus kerrii* (N50 = 1,280 bp; 720 bp) [27], *Quercus liaotungensis* (N50 = 1,180 bp; 695 bp) and *Quercus mongolica* (N50 = 1,189 bp; 704 bp) [28]. High-quality sequencing of the transcriptome will facilitate further genetic studies in this species.

A total of 46,338 (73.53%) unigenes were successfully annotated in at least one database (GO, KO, KOG, NR, NT, PFAM and SwissProt). The availability of annotated unigenes provided an important resource for studying various aspects of plant biology which can gain insights into the genetic underpinnings of various physiological and developmental processes in plants. These also provided a reference for studying plant hormone signal transduction and a critical foundation for quality character. All these various annotations and classifications generated from the analysis of *C*. *carlesii* unigenes offer useful resources for investigating the biological functions of specific unigenes of this species. A total of 16,674 unigenes failed to show matches to any functional annotation, which might be attributed to the short sequence length or the currently limited annotation of *Castanopsis* and related species in the database [29]. In addition, this may indicate that the vegetative and reproductive growth of *C*. *carlesii* involves many unique processes and pathways. The species-based annotations of unigenes showed that *C*. *carlesii* was closely related to *Q*. *suber*, rather than *Castanopsis* species, which was obviously inconsistent with taxonomic studies. This contradiction is likely due to the lack of genomic and transcriptome resources that are available for the related *Castanopsis* species in the publicly available databases.

### SSR markers in the transcriptome of *C*. *carlesii*

Transcriptome sequences can be used to develop EST-SSR markers that facilitate genetic diversity analysis and marker-assisted selection research. Among 63,012 unigenes, 31,459 potential EST-SSRs and 20,055 gene-based SSR marker were identified in 18,690 unigenes, with one SSR locus approximately per 2 kb. These values were relatively comparable to those reported for *Larix gmelinii* (one SSR per 2.87 kb) [11] and *Arachis hypogaea* (one SSR per 3.3 kb) [30], but higher than those reported for *Pinus dabeshanensis* (one SSR per 23.08 kb) [31], *Torreya grandis* (one SSR per 25.9 kb) [32] and *Zingiber officinale* (one SSR per 25.2 kb) [33]. Such divergence in the frequency of SSR motifs could be explained by the species differences, SSR search criteria, datasets sizes, and the mining tools used [34]. Excluding mono-nucleotide repeats, di-nucleotides were found to be the most abundant repeats, which was similar to studies conducted in other plants [35–38]. Additionally, the AG/CT motif was the most abundant dinucleotide repeat, and the CG/CG motif was the least abundant. This may be caused by the methylation of cytosine, which would inhibit transcription to some extent in plants [39,40]. Twenty percent of sequences were found to have SSRs in their CDS region, in contrast to 80% in UTRs. A similar phenomenon was observed in Chinese sweetgum [41]. This lower ratio of SSRs in CDS region is a result of selection pressure against open reading frame (ORF) changes in CDS regions that could cause potential frameshift mutations [42].

### Validation of EST-SSR markers

Forty-nine primer pairs (49%) successfully produced clear amplicons of the expected sizes among 100 randomly selected EST-SSR primer pairs. Twenty (20%) polymorphic EST-SSR markers were identified among eight individuals from different populations. This rate was comparable to the rates reported for *Chrysanthemum nankingense* (20%) [43], lower than those documented for *Juglans mandshurica* (30.8%) [44] and *Styrax japonicus* (35%) [45], and higher than the rates reported for *Neolitsea sericea* (16.03%) [40] and *Tephroseris furusei* (12%) [46]. Polymorphisms were verified using 25 samples obtained from *C*. *carlesii* in Qimen, Anhui. A total of 119 alleles were detected, with a mean number of alleles (*Na*) of 5.95 per locus and a mean polymorphism information content (*PIC*) of 0.6125, which represented a high level. The *PIC* value reflects the allelic diversity and was used to evaluate the level of information provided [47]. These results showed high levels of cross-amplification in the *Castanopsis* genus members, *C*. *eyrei* and *C*. *jucunda* (100%), *C*. *sclerophylla* and *C*. *lamontii* (95%). The transferability efficiency of the same set of markers slightly decreased to 90% in *C*. *fargesii*, owing to the greater phylogenetic distance that exists between *C*. *fargesii* and the other five species of *Castanopsis* [48]. The transferability ratios were higher than those obtained in *Rhododendron* (58.33–83.33%) [49] and *Elymus* (49.11%) [50]. Thus, the high transferability across part of this genus in the present study will provide valuable sequence resources for molecular marker development for *Castanopsis* species. The 20 newly developed EST-SSR markers can be applied to analyze the population genetics and marker-assisted selection breeding of *C*. *carlesii* and related species in the future. The genetic information provided by the results of population genetic will be crucial for understanding the variability within population, and differentiation among populations, the species evolutionary history and formulating conservation strategies. The marker-assisted selection breeding can significantly accelerate the breeding process and improve the efficiency of selecting individuals with desirable traits.

## Conclusions

In this study, we analyzed the transcriptome of *C*. *carlesii* and developed EST-SSR markers. A total of 149,380,224 clean reads and 63,012 nonredundant unigenes with a mean length of 1,034 bp were obtained. These 63,012 unigenes were assembled and annotated based on sequence similarity searches in the Nr (56.12%), Nt (43.22%), KO (22.43%), SwissProt (50.11%), PFAM (48.51%), KO (22.43%), and GO (48.51%) databases. A total of 31,459 potential SSRs were identified in 18,690 unigenes. We randomly selected 100 EST-SSRs for the evaluation of the polymorphism, and 20 primer pairs revealed abundant polymorphism. Polymorphisms were verified in the Qimen population, which detected 119 alleles and a mean polymorphism information content (*PIC*) of 0.6125. Among these 20 newly developed EST-SSR markers, sixteen primer pairs showed successful amplification in all five *Castanopsis* species (80%), and the transferability ratios ranged from 90% to 100%. These EST-SSR markers provide powerful molecular tools for analyzing the population genetics and population structure of *C*. *carlesii* and in *Castanopsis* species.

## Supporting information

S1 Fig Sequence length distribution of all assembled unigenes and transcripts in *C. carlesii*. The x-axis indicates the sizes of all unigenes and transcripts, and the y-axis indicates the numbers of sequences with a certain length.
(TIF)

S2 Fig Annotation information distribution in seven databases in *C. carlesii*. The x-axis indicates the number of unigenes, and the y-axis indicates seven databases.
(TIF)

S3 Fig Species distribution classification map based on the NR database. (TIF)

S4 Fig Partial capillary electrophoresis profiles of primer pairs CC57, CC45 and CC86. (TIF)

S1 Table Sampling information for *Castanopsis* species *used* in this study. (DOCX)

S2 Table Annotation information from seven databases for *C. carlesii*. (DOCX)

S3 Table Summary of Gene Ontology (GO) term assignments for the *C. carlesii* transcriptome (Level 2). (DOCX)

S4 Table KOG annotation of *C. carlesii*. (DOCX)

S5 Table Summary of the KEGG pathway annotation of *C. carlesii*. (DOCX)

S6 Table Primer details of a total of 100 SSR markers for validation. (XLS)
