# Supplementary figures and images for: Development of EST-SSRs based on the transcriptome of Castanopsis carlesii and cross-species transferability in other Castanopsis species
Source: PLoS One. 2023 Jul 20;18(7):e0288999. doi: 10.1371/journal.pone.0288999 (PMC10358944; doi:10.1371/journal.pone.0288999)

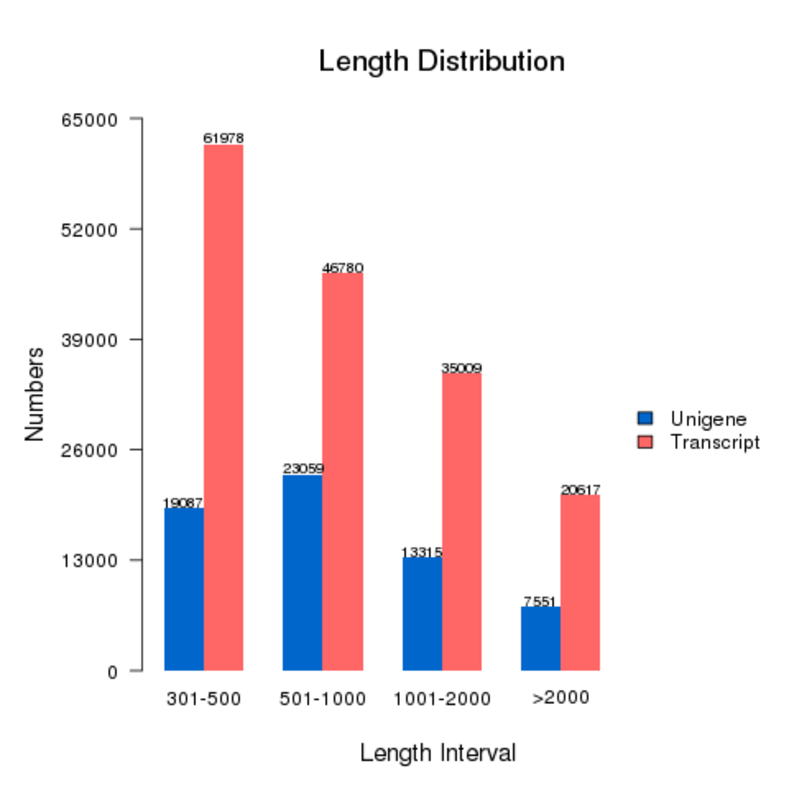

Supplement: S1 Fig — The x-axis indicates the sizes of all unigenes and transcripts, and the y-axis indicates the numbers of sequences with a certain length. (TIF) [file pone.0288999.s001.tif]

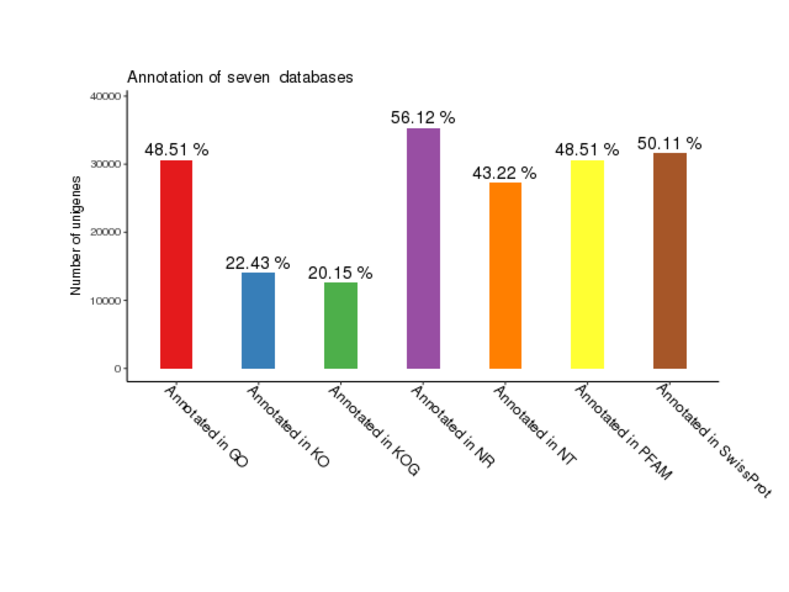

Supplement: S2 Fig — The x-axis indicates the number of unigenes, and the y-axis indicates seven databases. (TIF) [file pone.0288999.s002.tif]

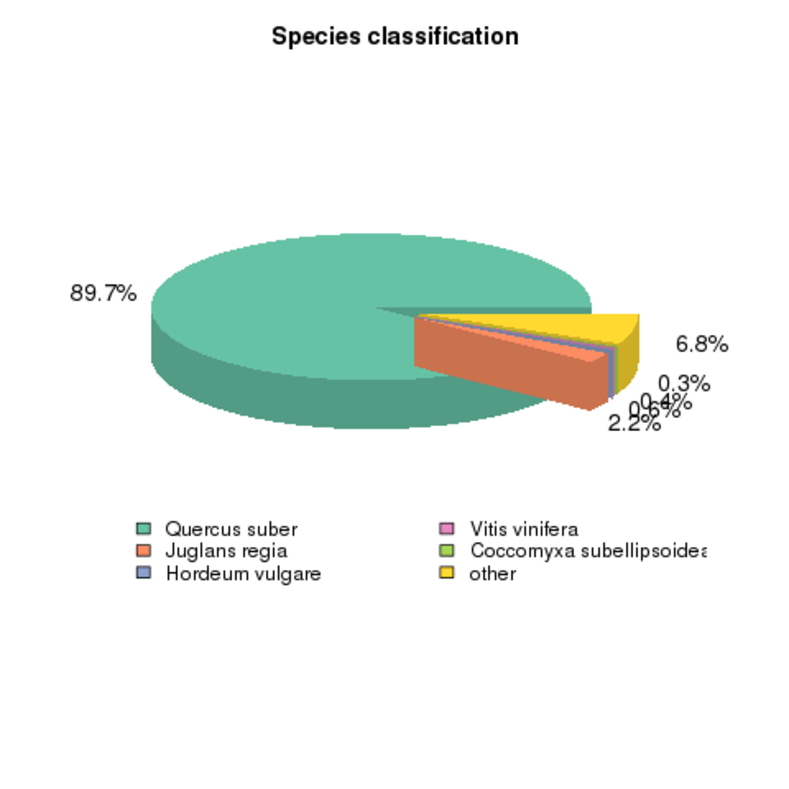

Supplement: S3 Fig — (TIF) [file pone.0288999.s003.tif]

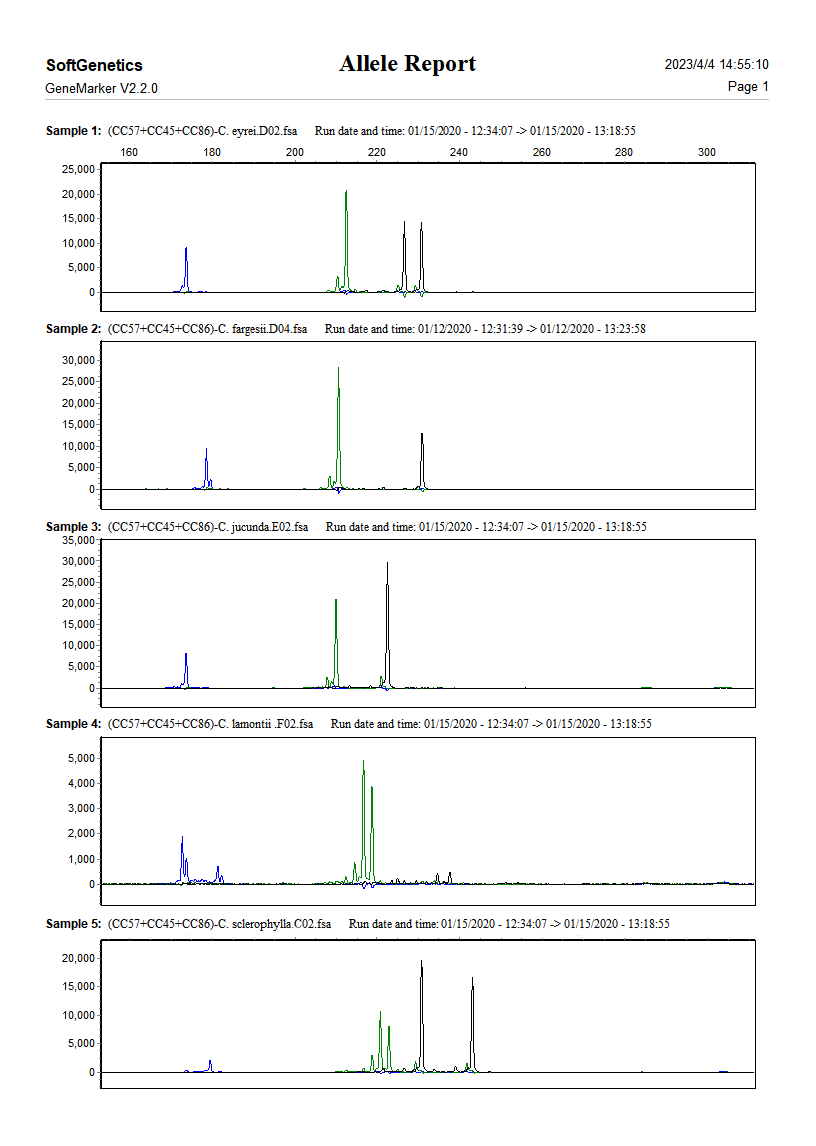

Supplement: S4 Fig — (TIF) [file pone.0288999.s004.tif]
