# Supplementary material for: Development of EST-SSRs based on the transcriptome of Castanopsis carlesii and cross-species transferability in other Castanopsis species
Source: PLoS One. 2023 Jul 20;18(7):e0288999. doi: 10.1371/journal.pone.0288999 (PMC10358944; doi:10.1371/journal.pone.0288999)
Supplement: S1 Table — (DOCX) [file pone.0288999.s005.docx]

**S1 Table.** Sampling information of *Castanopsis* species used in this study*.*

| Species | Sampling Location | Number of Sample | Longitude (E) | Latitude (N) |
| --- | --- | --- | --- | --- |
| *Castanopsis carlesii* | Qimen, Anhui | 25 | 117.27 | 29.98 |
| *Castanopsis carlesii* | Chongyi, Jiangxi | 1 | 114.26 | 25.79 |
| *Castanopsis carlesii* | Longnan, Jiangxi | 1 | 114.43 | 24.57 |
| *Castanopsis carlesii* | Lianping, Guangdong | 1 | 114.45 | 24.47 |
| *Castanopsis carlesii* | Dingnan, Jiangxi | 1 | 115.14 | 24.92 |
| *Castanopsis carlesii* | Quannan, Jiangxi | 1 | 114.46 | 24.61 |
| *Castanopsis carlesii* | Hengyang, Hunan | 1 | 112.69 | 27.25 |
| *Castanopsis carlesii* | Xinfeng, Guangdong | 1 | 114.12 | 24.13 |
| *Castanopsis clerophylla* | Nanchang, jiangxi | 1 | 115.82 | 28.75 |
| *Castanopsis lamontii* | Ganzhou，Jiangxi | 1 | 114.42 | 24.55 |
| *Castanops fargesii* | Ganzhou，Jiangxi | 1 | 114.42 | 24.55 |
| *Castanopsis eyrei* | Ganzhou，Jiangxi | 1 | 114.42 | 24.55 |
| *Castanopsis jucunda* | Nanchang, jiangxi | 1 | 115.82 | 28.75 |
