# Supplementary material for: Development of EST-SSRs based on the transcriptome of Castanopsis carlesii and cross-species transferability in other Castanopsis species
Source: PLoS One. 2023 Jul 20;18(7):e0288999. doi: 10.1371/journal.pone.0288999 (PMC10358944; doi:10.1371/journal.pone.0288999)
Supplement: S2 Table — (DOCX) [file pone.0288999.s006.docx]

**S2 Table.** The annotation information of seven databases in *C. carlesii*

| Annotation information | Number of Unigenes | Percentage (%) |
| --- | --- | --- |
| Annotated in GO | 30570 | 48.51 |
| Annotated in KO | 14134 | 22.43 |
| Annotated in KOG | 12701 | 20.15 |
| Annotated in NR | 35366 | 56.12 |
| Annotated in NT | 27238 | 43.22 |
| Annotated in PFAM | 30570 | 48.51 |
| Annotated in SwissProt | 31579 | 50.11 |
| Annotated in all Databases | 5559 | 8.82 |
| Annotated in at least one Database | 46338 | 73.53 |
| Total Unigenes | 63012 | 100 |
