# Supplementary material for: Development of EST-SSRs based on the transcriptome of Castanopsis carlesii and cross-species transferability in other Castanopsis species
Source: PLoS One. 2023 Jul 20;18(7):e0288999. doi: 10.1371/journal.pone.0288999 (PMC10358944; doi:10.1371/journal.pone.0288999)
Supplement: S3 Table — (DOCX) [file pone.0288999.s007.docx]

**S3 Table.** Summary of Gene Ontology (GO) term assignment for *C. carlesii* transcriptome (Level 2)

| GO type | GO Term (Level 2) | Gene number | Percent（%） |
| --- | --- | --- | --- |
| Biological Process | cellular process | 16637 | 54.42 |
|  | metabolic process | 15892 | 51.99 |
|  | single-organism process | 13060 | 42.72 |
|  | biological regulation | 5432 | 17.77 |
|  | regulation of biological process | 5075 | 16.60 |
|  | localization | 4858 | 15.89 |
|  | response to stimulus | 3714 | 12.15 |
|  | cellular component organization or biogenesis | 3431 | 11.22 |
|  | signaling | 1963 | 6.42 |
|  | multi-organism process | 1563 | 5.11 |
|  | multicellular organismal process | 693 | 2.27 |
|  | developmental process | 513 | 1.68 |
|  | reproduction | 423 | 1.38 |
|  | reproductive process | 407 | 1.33 |
|  | positive regulation of biological process | 296 | 0.97 |
|  | locomotion | 254 | 0.83 |
|  | negative regulation of biological process | 248 | 0.81 |
|  | immune system process | 180 | 0.59 |
|  | biological adhesion | 174 | 0.57 |
|  | growth | 34 | 0.11 |
|  | rhythmic process | 29 | 0.09 |
|  | cell killing | 28 | 0.09 |
|  | behavior | 25 | 0.08 |
|  | biological phase | 23 | 0.08 |
|  | detoxification | 11 | 0.04 |
|  | cell aggregation | 2 | 0.01 |
