# Supplementary material for: Development of EST-SSRs based on the transcriptome of Castanopsis carlesii and cross-species transferability in other Castanopsis species
Source: PLoS One. 2023 Jul 20;18(7):e0288999. doi: 10.1371/journal.pone.0288999 (PMC10358944; doi:10.1371/journal.pone.0288999)
Supplement: S4 Table — (DOCX) [file pone.0288999.s008.docx]

**S4 Table.** KOG annotation of *C. carlesii*

| KOG categories | KOG description | Number | Percent (%) |
| --- | --- | --- | --- |
| A | [A] RNA processing and modification | 584 | 4.60 |
| C | [C] Energy production and conversion | 1446 | 11.38 |
| B | [B] Chromatin structure and dynamics | 207 | 1.63 |
| E | [E] Amino acid transport and metabolism | 576 | 4.54 |
| D | [D] Cell cycle control, cell division, chromosome partitioning | 265 | 2.09 |
| G | [G] Carbohydrate transport and metabolism | 747 | 5.88 |
| F | [F] Nucleotide transport and metabolism | 161 | 1.27 |
| I | [I] Lipid transport and metabolism | 578 | 4.55 |
| H | [H] Coenzyme transport and metabolism | 233 | 1.83 |
| K | [K] Transcription | 453 | 3.57 |
| J | [J] Translation, ribosomal structure and biogenesis | 1950 | 15.35 |
| M | [M] Cell wall/membrane/envelope biogenesis | 100 | 0.79 |
| L | [L] Replication, recombination and repair | 236 | 1.86 |
| O | [O] Posttranslational modification, protein turnover, chaperones | 1876 | 14.77 |
| N | [N] Cell motility | 3 | 0.02 |
| Q | [Q] Secondary metabolites biosynthesis, transport and catabolism | 321 | 2.53 |
| P | [P] Inorganic ion transport and metabolism | 377 | 2.97 |
| S | [S] Function unknown | 555 | 4.37 |
| R | [R] General function prediction only | 1559 | 12.27 |
| U | [U] Intracellular trafficking, secretion, and vesicular transport | 686 | 5.40 |
| T | [T] Signal transduction mechanisms | 640 | 5.04 |
| W | [W] Extracellular structures | 9 | 0.07 |
| V | [V] Defense mechanisms | 70 | 0.55 |
| Y | [Y] Nuclear structure | 51 | 0.40 |
| Z | [Z] Cytoskeleton | 301 | 2.37 |
