# Supplementary material for: Development of EST-SSRs based on the transcriptome of Castanopsis carlesii and cross-species transferability in other Castanopsis species
Source: PLoS One. 2023 Jul 20;18(7):e0288999. doi: 10.1371/journal.pone.0288999 (PMC10358944; doi:10.1371/journal.pone.0288999)
Supplement: S5 Table — (DOCX) [file pone.0288999.s009.docx]

**S5 Table.** Summary of KEGG pathways annotation of *C. carlesii*

| Pathway Hierarchy1 | Pathway Hierarchy2 | Gene number | Percentage(%) |
| --- | --- | --- | --- |
| Cellular Processes | Transport and catabolism | 801 | 6.00 |
| Environmental Information Processing | Membrane transport | 43 | 0.32 |
|  | Signal transduction | 269 | 2.02 |
| Genetic Information Processing | Folding, sorting and degradation | 984 | 7.38 |
|  | Replication and repair | 180 | 1.35 |
|  | Transcription | 525 | 3.94 |
|  | Translation | 2015 | 15.11 |
| Metabolism | Amino acid metabolism | 1004 | 7.53 |
|  | Biosynthesis of other secondary metabolites | 418 | 3.13 |
|  | Carbohydrate metabolism | 1798 | 13.48 |
|  | Energy metabolism | 896 | 6.72 |
|  | Glycan biosynthesis and metabolism | 152 | 1.14 |
|  | Lipid metabolism | 806 | 6.04 |
|  | Metabolism of cofactors and vitamins | 369 | 2.77 |
|  | Metabolism of other amino acids | 482 | 3.61 |
|  | Metabolism of terpenoids and polyketides | 373 | 2.80 |
|  | Nucleotide metabolism | 326 | 2.44 |
|  | Overview | 1417 | 10.62 |
| Organismal Systems | Environmental adaptation | 481 | 3.61 |
| Total |  | 13339 | 100 |
